# Difference in the metabolic characteristics of chronic obstructive pulmonary disease patients and healthy adults

**DOI:** 10.1097/MD.0000000000021302

**Published:** 2020-07-24

**Authors:** Yongliang Jiang, Hantong Hu, Xiaoyu Li, Xiaofen He, Xiaomei Shao, Jiali Lou, Yajun Zhang, Yuanyuan Wu, Jianqiao Fang

**Affiliations:** a Department of Neurobiology and Acupuncture Research, The Third Clinical Medical College, Zhejiang Chinese Medical University, Key Laboratory of Acupuncture and Neurology of Zhejiang Province, Hangzhou; b The Third Affiliated Hospital of Zhejiang Chinese Medical University, Hangzhou City, Zhejiang Province, China.

**Keywords:** chronic obstructive pulmonary disease, functional near infrared spectroscopy, meridians, moxibustion, oxygenation metabolism

## Abstract

**Introduction:**

By detecting the metabolic difference of the Heart and Lung meridians, the present study aims to investigate the specificity of different meridians and verify whether functional near infrared spectroscopy is validated as an add-on technique to assist diagnosis of chronic obstructive pulmonary disease (COPD).

**Methods and analysis:**

The Lung and Heart meridians are chosen as the target for comparison; accordingly, 120 eligible participants will be included and divided into the COPD group, healthy control group, and healthy intervention group. Functional near infrared spectroscopy will be adopted to measure the metabolic characteristics of the Heart and Lung meridians. On one hand, the specificity of the meridian-visceral association will be investigated by comparing the metabolic difference in the Heart and Lung meridians between the healthy control group and COPD group. On the other hand, the specificity of site-to-site association will be determined by comparing the metabolic change between the 2 meridians that induced by moxibustion in the Heart meridian and Lung meridian, respectively, in the healthy control group. The primary outcome will be regional oxygen saturation of corresponding regions along the Heart and Lung meridians.

**Trial registration:**

ClinicalTrials.gov NCT04046666.

## 1 Introduction

As a major part of complementary and alternative medicine, acupuncture is frequently used alone, or as an adjunctive therapy to treat a wide range of diseases. In the past years, increasing high-quality studies of acupuncture trials have been published in influential academic journals, which indicate the efficacy of acupuncture for treating various kinds of diseases, such as chronic functional constipation,^[1]^ migraine prophylaxis,^[2]^ chronic stable angina,^[3]^ and postprandial distress syndrome.^[4]^ Thus, acupuncture is receiving increasing attention and acceptance worldwide.^[5]^

Nevertheless, as the principle theory to guild acupuncture manipulations for thousands of years, the validation of meridian theory and the existence of meridians, remain unrecognized by many researchers due to lack of evidence.^[6,7]^ Based on traditional Chinese medicine, meridians are passageways of “qi” and “blood” and connect the body surface with internal organs, thus acting as contacts, adjustments, and response systems.

After a long period of investigations, despite that the anatomical structure of meridians remains unclear, the existence of the meridian phenomenon has been confirmed.^[8]^ And multiple biophysical techniques have been adopted to verify the biological characteristics of meridians, such as the electrical, acoustic, optical, and magnetic characteristics.^[9–12]^ Nevertheless, the majority of previous studies were designed to investigate the correlation between 1 meridian and its corresponding organ, or the difference between meridians and non-meridians, the correlation and specificity between different meridians are seldom explored. Moreover, apart from the aforementioned biological properties, metabolic characteristic is also one of the major characteristics of meridians and is relatively a new research hotspot in meridian studies, which could be measured by functional near infrared spectroscopy (fNIRS). As an established approach to monitor tissue oxygenation metabolism, fNIRS is becoming a popular tool in acupuncture studies.^[13]^

In addition, it is revealed that low oxygen saturation could be observed in peripheral vessels of chronic obstructive pulmonary disease (COPD) patients, but it remains unclear whether the distribution regions of the Lung meridians is characterized by abnormal oxygen metabolism in relative to distribution regions of the other meridians or non-meridians.

Thus, the present study will use fNIRS to detect the metabolic characteristics of meridians. We choose the Lung and Heart meridians as the target for comparison, accordingly, patients with COPD and healthy subjects will be included. Based on comparison of metabolic characteristics between these 2 meridians, the present study aims to investigate the association and specificity of different meridians. Besides, it will verify whether fNIRS is validated as an add-on technique to assist diagnosis of COPD. The findings of this study are expected to add scientific evidence for traditional meridian theory and promote the selection of acupoints in practice.

## 2 Methods and design

### 2.1 Study design

**Figure 1:**
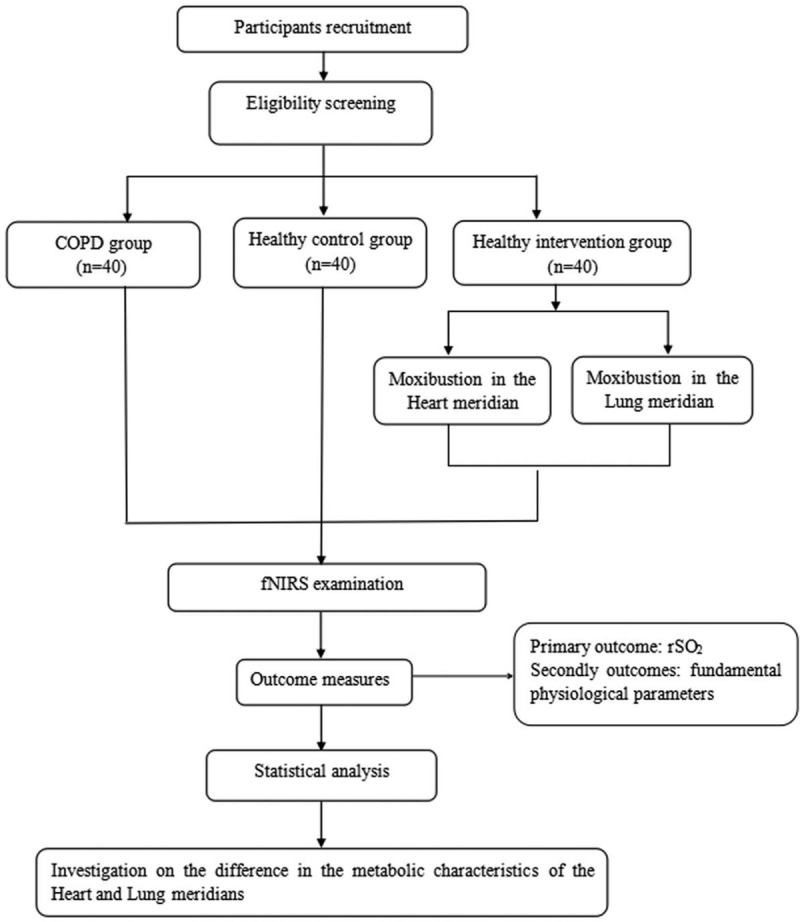
Study design flowchart. COPD = chronic obstructive pulmonary disease, fNIRS = functional near infrared spectroscopy, rSO_2_ = regional oxygen saturation.

This study is designed as a prospective and clinical controlled trial. Eligible participants will be divided into the COPD group, healthy control group, and healthy intervention group. The study flowchart is presented in Figure 1 and the schedule for participant screening, interventions, and outcome assessments is shown in Table 1. The report of this protocol will strictly follow the guideline of the Standards for Reporting Interventions in Clinical Trials of Acupuncture.^[14]^

**Table 1:**
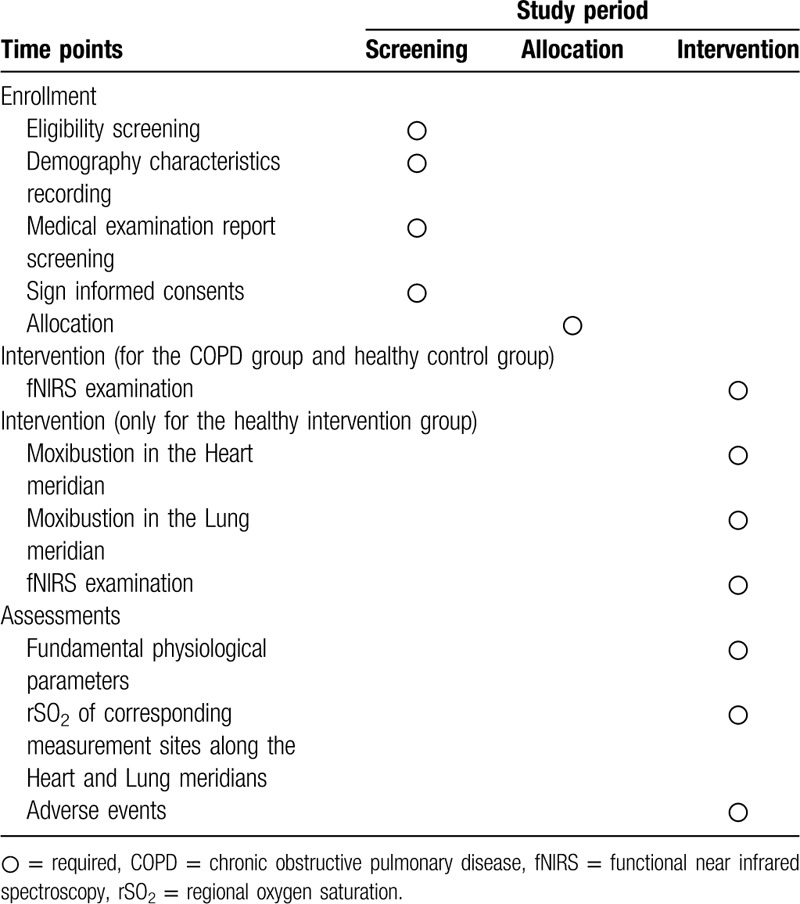
Trial schedule for enrollment, intervention, and assessments.

### 2.2 Sample size calculation

This study is a clinical trial that aims to measure the biological characteristics of meridians in COPD patients and healthy adults. Compared with common clinical trials that intend to evaluate the effectiveness of a specific therapy, there is no consensus on the standardization of sample size estimation for this kind of study. Thus, the sample size estimation is mainly according to similar trials^[15,16]^ and our preliminary study.^[12]^ Finally, the total number of participants is 120, and they will be divided into the COPD group, healthy control group, and healthy intervention group in a 1:1:1 ratio.

### 2.3 Subjects

The subjects of this study consist of COPD patients and healthy volunteers.

#### 2.3.1 Inclusion criteria

##### 2.3.1.1 Inclusion criteria of COPD patients

(1) Patients should satisfy the COPD diagnostic criteria, and the level of COPD is GOLD 2 or 3 according to results of pulmonary function testing;
(2) Patients are in the stable phase of COPD;
(3) 35 ≤ age ≤ 75 years, gender is not limited;
(4) Patients have clear consciousness and can communicate with researchers normally;
(5) Patients can understand the study protocol and agree to sign written informed consents.

##### 2.3.1.2 Inclusion criteria for the healthy volunteers

(1) Healthy volunteers can provide a recent medical examination report, which can verify that they do not have major neurological, cardiovascular, respiratory, digestive, urinary, endocrine, and hematological diseases;
(2) Age ≥20 years, gender is not limited;
(3) Participants have clear consciousness and can communicate with researchers normally;
(4) Participants can understand the study protocol and agree to sign written informed consents.

#### 2.3.2 Exclusion criteria

##### 2.3.2.1 Exclusion criteria of the COPD group

(1) Patients cannot satisfy the COPD diagnostic criteria, or COPD patients in acute exacerbation;
(2) COPD patients have other major lung diseases, such as bronchiectasis, bronchial asthma, pneumothorax, chest trauma, and lung tumors;
(3) Patients have concomitant heart diseases;
(4) Patients have serious concomitant diseases in major systems and could not control them effectively at present, such as major diseases in the nervous, respiratory, digestive, urinary, and hematological system;
(5) Patients have mental diseases, history of drug abuse and alcohol dependence;
(6) Patients are lactating or pregnant;
(7) Patients are taking part in other studies.

##### 2.3.2.2 Exclusion criteria of the healthy volunteers

(1) Participants have mental illness, history of drug abuse and alcohol dependence;
(2) Participants are lactating or pregnant;
(3) Participants are taking part in other studies.

### 2.4 Recruitment procedures

All participants will be enrolled from the Third Affiliated Hospital of Zhejiang Chinese Medical University. Our study will be advertised on the Internet and on posters in hospitals. Researchers will explain the aim, benefits, and potential risks of the study to participants in details. After signing written informed consents, all participants will be assessed by researchers to determine whether they can be included based on the inclusion and exclusion criteria.

### 2.5 Blinding

Due to the nature of an open-labeled trial, we will not blind the manipulators, participants, and outcome assessors, only blinded statistical analysis will be adopted by employing statisticians who are unaware of the study protocol.

### 2.6 Experimental procedures

Participants will be requested to refrain from consuming stimulating drinks (eg, tea, alcohol, coffee) and smoking on the day for fNIRS examination. Besides, exercise and food will be forbidden within 1 hour before fNIRS examinations.

#### 2.6.1 fNIRS examination environment

An experimental room will be set up for fNIRS examinations. The temperature for the examination environment will be controlled within 24°C ± 1°C. The relative humidity will be controlled between 30% and 50%. There is no direct sunlight and obvious air convection in the room.

#### 2.6.2 Procedures for the fNIRS examinations and moxibustion

A 4-channel Oximeter (INVOS 5100C, Somanetics Corp., Troy, MI) will be used to detect the metabolic property of the Heart and Lung meridians. Before fNIRS examinations, all participants will stabilize for 15 minutes. They will also be requested to keep normal breathe, maintain still, and avoid movement of limbs during fNIRS measurement. After ripping adhesive tapes, the fNIRS probes will be attached to corresponding measurement sites synchronously. Oxygen saturation of corresponding regions along the Heart and Lung meridians will be monitored.

**Figure 2:**
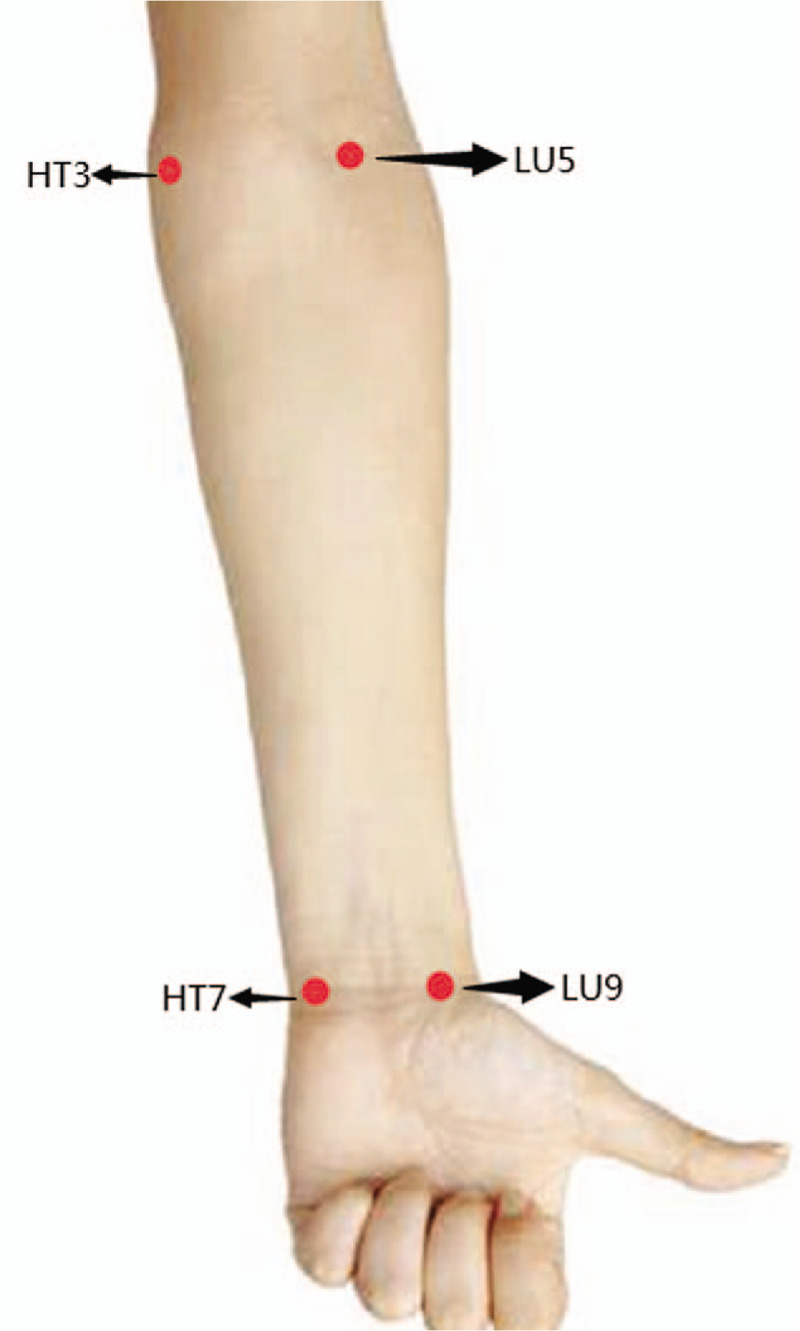
The fNIRS measurement sites of the COPD group and healthy control group. COPD = chronic obstructive pulmonary disease, fNIRS = functional near infrared spectroscopy.

**Figure 3:**
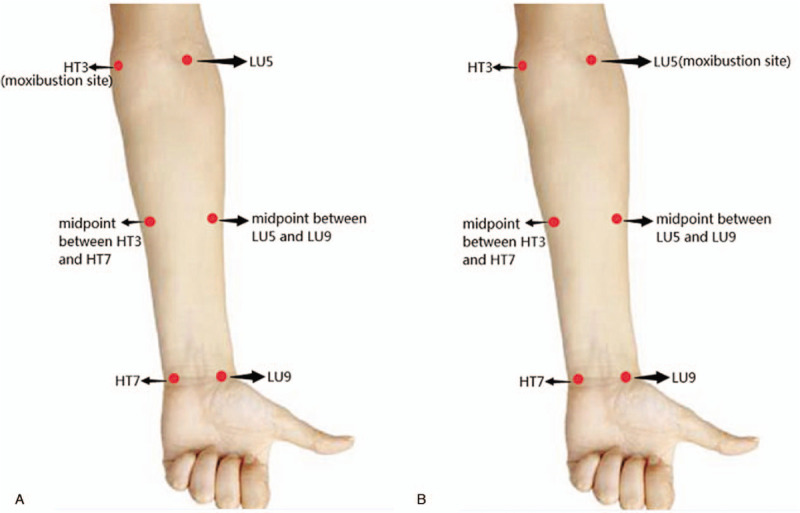
The intervention procedure of the healthy intervention group. (A) Moxibustion site of the Heart meridian and the fNIRS measurement sites of the Heart and Lung meridians; (B) Moxibustion site of the Lung meridian and the fNIRS measurement sites of the Heart and Lung meridians. fNIRS = functional near infrared spectroscopy.

(1) Healthy control group and COPD group
  The fNIRS probes will be attached to 4 measurement sites synchronously (as shown in Fig. 2) to monitor oxygen saturation for 10 minutes, including acupoint HT3 and HT7 and of the Heart meridian, acupoint LU5 and LU9 and of the Lung meridian.
(2) Healthy intervention group
  Moxibustion will be performed in the Heart meridian and Lung meridian, respectively. The washout period between the 2 moxibustion sessions is more than 1 day. The procedures for intervention and the measurement places of the fNIRS probes are displayed in Figure 3.

1) Moxibustion in the Heart meridian: a moxa stick is ignited and inserted into a homemade holder to adjust appropriate height and angle, moxibustion is applied at acupoint HT3 of the Heart meridian for 15 minutes. The fNIRS probes will be attached to 3 measurement sites during moxibustion, including the midpoint of the Heart meridian along the left forearm (i.e. the midpoint between HT7 and HT3), the midpoint of the Lung meridian along the forearm (i.e. the midpoint between LU5 and LU9), and LU5 of the Lung meridian. The procedures for intervention and the measurement places of the fNIRS probes are shown in Figure 3A. The measurement time points consist of 5 minutes before moxibustion, 15 minutes during moxibustion, and 5 minutes after removing moxibustion.
2) Moxibustion in the Lung meridian: moxibustion is applied at acupoint LU5 of the Lung meridian. The moxibustion procedure, fNIRS examinations and assessment time points are consistent with 1). The fNIRS probes will monitor oxygen saturation in 3 measurement sites synchronously during moxibustion, including the midpoint of the Lung meridian along the forearm (ie, the midpoint between LU5 and LU9), and the midpoint of the Heart meridian along the left forearm (ie, midpoint between HT3 and HT7, and HT3 of the Heart meridian. The procedures for intervention and the measurement places of the fNIRS probes are displayed in Figure 3B.

#### 2.6.3 Acupoint locations and fNIRS measurement sites

(1) “Shenmen” (HT7): On the anteromedial aspect of the wrist, radial to the flexor carpi ulnaris tendon, on the palmar wrist crease.
(2) “Shaohai” (HT3): On the anteromedial aspect of the elbow, just anterior to the medial epicondyle of the humerus, at the same level as the cubital crease.
(3) “Taiyuan” (LU9): On the anterolateral aspect of the wrist, between the radial styloid process and the scaphoid bone, in the depression ulnar to the abductor pollicis longus tendon.
(4) “Chize” (LU5): On the anterior aspect of the elbow, at the cubital crease, in the depression lateral to the biceps brachii tendon.
(5) The midpoint of the Heart meridian along the forearm: the midpoint between HT3 and HT7.
(6) The midpoint of the Lung meridian along the forearm: the midpoint between LU5 and LU9.

#### 2.6.4 Concomitant treatments

Participants in the COPD group will maintain current treatments during study. If new medications or therapies are added throughout the study, they should be recorded in details.

Besides, participants in the healthy group or healthy control group are not permitted to take any medications during study. If they suffer from sudden diseases and medications or other treatments are applied for emergency, corresponding information should be recorded and researchers will determine whether they will be suspended from the trial.

### 2.7 Outcomes

The primary outcome will be regional oxygen saturation of the aforementioned sites that located in the Heart and Lung meridians. Second outcomes will be fundamental physiological parameters measured before and immediately after fNIRS examinations, including body temperature, heart rate, breathing rate, and blood pressure.

### 2.8 Safety evaluation

Adverse events that occur during the trial will be documented and assessed by researchers. If serious adverse events occur, they should be reported to the ethics committee by researchers immediately and remedial measures should be adopted in time.

### 2.9 Ethics approval

This study has been approved by the Ethics Committee of the Third Affiliated Hospital of Zhejiang Chinese Medical University (approval number. ZSLL-KY-2019-001F-01). Informed consent will be signed by the participants before they are included in the study.

### 2.10 Trial registration

The trial has been registered at clinical trials (identification code NCT04046666).

### 2.11 Data collection and statistical analysis

SPSS 17.0 for Windows (SPSS Inc., Chicago, IL) will be used to perform statistical analysis. For data expression, numeric data in normal distribution will be presented as mean and standard deviations, and data in skewed distribution will be presented as median and interquartile ranges. For statistical analysis methods, change in continuous variables at different time points will be analyzed by repeated measures analysis of variance. The changes within the groups will analyzed via the paired samples *t*-test, whereas differences between the groups will be compared via the independent samples *t*-test. If data is in skewed distribution, nonparametric test will be applied to perform within-group or between-group comparison. Statistical significance is defined as a *P*-value less than .05.

### 2.12 Quality control

This protocol has been revised based on suggestions of experienced acupuncturists before conducting the trial. All researchers will receive intensive training courses to master the standard operating procedures. Procedures of fNIRS examinations will be trained by specific experts in this field. Case report forms will be verified by monitors at regular intervals throughout the study. In addition, economic compensation will be adopted to improve participant compliance and reduce dropouts.

## 3 Discussion

Systematic studies on meridians in China could date back to the 1950s.^[8]^ After a long period of investigations, despite that the physical essence of meridians remains a mystery, a great number of studies reveal various distinctive biological properties of meridian paths as relative to non-meridian regions, such as low electrical resistance,^[17]^ increased temperature,^[18]^ vigorous blood perfusion,^[19]^ low hydraulic resistance^[20]^ along meridians. Apart from these biological properties of meridians, metabolic characteristic is relatively a new research hotspot and needs further investigation. Metabolic characteristic is one of the major properties of meridians and acupoints. The specific metabolic characteristic of tissues underneath the skin surface has certain correlations with meridians. For example, SX Zheng et al^[21]^ found that in physiological condition, the oxygen metabolism along the pericardium meridian was significantly more vigorous than its bilateral non-meridian paths. Given that fNIRS is a recently developed spectroscopic technique to monitor oxygenation metabolism and has advantages of noninvasiveness, ease of use, dynamic detection, and high temporal resolution,^[22]^ it is receiving increasing attention in acupuncture trials.^[23,24]^ Because near-infrared light can penetrate into the tissue deeply to detect change in tissue oxygenation very sensitively,^[25]^ the oxygen-dependent absorption of light by haemoglobin enables the calculation of relative changes of specific parameters reflecting the condition of oxygenation metabolism by fNIRS,^[26]^ such as regional oxygen saturation that assessed in the present study. Oxygen saturation results from a dynamic balance between O_2_ supply and consumption in arteriolar, capillary and venular beds and it is generally regarded that local oxygen saturation has close correlation to regional energy metabolism and blood perfusion.^[27]^

Nevertheless, the majority of previous studies were designed to investigate the correlation between 1 meridian and its corresponding organs, or the difference between meridians (or acupoints) and non-meridians (or non-acupoints). Investigation on the correlation and specificity between 1 meridian and the other meridian is very rare.^[28]^ Thus, there are several highlights and advantages of the present study.

First, as far as we are concerned, the present study is the first case-controlled trial that designed to investigate the correlation and specificity between 2 different meridians (ie, the Heart meridian and the Lung meridian) by using an objective assessment technique, fNIRS. We propose a hypothesis that during the change from the physiological to pathological state, the metabolic characteristics of the affected meridian will change synchronously. And significant difference in the metabolic characteristics might be detected between the affected meridian (ie, the Lung meridian for COPD patients) and the unaffected meridian (ie, the Heart meridian for COPD patients). Meanwhile, in the healthy control group, we put forward a hypothesis that the stimulated meridian will present with more significant change in the metabolic characteristics in relative to the non-stimulated meridian. Given that a previous study revealed that thermal stimulation could significant increase local oxygen saturation,^[24]^ in this study, moxibustion is selected as the stimulation modality. Specifically, on the one hand, the specificity of meridian-visceral association will be explored based on a metabolic comparison in the Heart and Lung meridians between the healthy control group and the CPPD group. On the other hand, the specificity of site-to-site association will be determined based on the comparison of the metabolic change between the 2 meridians that induced by moxibustion in the Heart meridian and Lung meridian, respectively, in the healthy control group.

Second, based on comparison upon the metabolic differences of the Heart and Lung meridians in healthy adults and COPD patients, it will verify whether fNIRS is validated as an add-on technique to assist diagnosis of specific diseases that affects oxygen metabolism in tissue, such as COPD. Previous studies revealed that low oxygen saturation could be detected in peripheral vessels in COPD patients.^[29–31]^ Similarly, based on the theory of meridian-viscera association in traditional Chinese medicine, pathological changes of internal organs will manifest in the external regions or acupoints that belongs to the diseased meridian, and this phenomenon is confirmed by a number of modern studies.^[32,33]^ Nevertheless, it remains unclear whether the distribution regions of the Lung meridians present with abnormal oxygen metabolism in relative to distribution regions of the other meridians or non-meridian paths, which will be verified by the findings of our study.

Third, various kinds of measures will be made to minimize the confounding factors that affect the tissue oxygen metabolism. In details, an experimental room with controlled temperature and humidity is set up and participants are asked to rest for a long period to adapt to the environment. All fNIRS examinations are performed at about the same period of the day. Meanwhile, stimulating food or drink, and exercise are restricted before fNIRS examinations. Fundamental physiological parameters will be measured before and immediately after fNIRS examinations, with the aim to eliminate the possibility that the change of oxygen metabolism is resulted from significant change of fundamental physiological parameters in normal state. Moreover, in order to minimize individual differences, the ratio of participant gender is controlled as 1:1 and the age between the COPD group and the healthy group will try to be matched as much as possible.

Nevertheless, several limitations of the present study should be emphasized. First, due to the exploratory nature as a pilot trial, the study involves a small sample size. Nevertheless, this pilot study will determine whether the feasibility of the study design is validate and provide data for a large scale study in the future. Second, although various measures will be adopted to minimize external and internal factors that affect tissue oxygen metabolism, given that individual differences might be inevitable in some cases, which will reduce the reliability of fNIRS results in a certain extent.

## 4 Conclusions

By comparing the metabolic characteristics of the Heart and Lung meridians via fNIRS, the present case-controlled trial aims to investigate the association and specificity of different meridians. Besides, it will verify whether fNIRS is validated as an add-on technique to assist diagnosis of COPD. The findings of this study are expected to bring scientific evidence for traditional meridian theory and promote the selection of acupoints in clinical practice.

## Author contributions

Jianqiao Fang, Yongliang Jiang, and Hantong Hu designed the trial protocol and drafted the manuscript. Yuanyuan Wu and Xiaofen He planned a data analysis solution. Xiaoyu Li, Jiali Lou, Xiaomei Shao, and Yajun Zhang participated in recruitment, fNIRS examination, and data collection of participants. All the authors have read, revised, and approved this version of the manuscript.
